# Effectiveness of extracorporeal shock wave therapy on functional ability in grade IV knee osteoarthritis – a randomized controlled trial

**DOI:** 10.1038/s41598-024-67511-x

**Published:** 2024-07-17

**Authors:** Arshed CP, Kavitha Jayaraman, Reem Abdullah Babkair, Shibili Nuhmani, Alvina Nawed, Masood Khan, Ahmad H. Alghadir

**Affiliations:** 1 Department of Physiotherapy, AWH Special College, Kozhikode, Affiliated to Kerala University of Health Sciences, Kozhikode, India; 2 Physiotherapy Department, Alhada Armed Forces Hospital, Alhada, Taif, Saudi Arabia; 3 Department of Physical Therapy, College of Applied Medical Sciences, Imam Abdulrahman Bin Faisal University, Dammam, Saudi Arabia; 4 Department of Rehabilitation Science, Jamia Hamdard, New Delhi, India; 5 Rehabilitation Research Chair, Department of Rehabilitation Sciences, College of Applied Medical Sciences, King Saud University, Riyadh, Saudi Arabia

**Keywords:** Physical therapy, Lower extremity functional scale score, Ultrasound therapy, Exercises, Quadriceps, Rehabilitation, Osteoarthritis

## Abstract

Extracorporeal shockwave therapy (ESWT) is a non-invasive physical therapy intervention that has emerged in the recent past to address the upswing of osteoarthritis (OA). However, insufficient evidence is present to prove the efficacy of ESWT on grade IV knee osteoarthritis (KOA). The present study aimed to examine the effects of ESWT on functional ability in patients suffering from grade IV KOA. Thirty volunteers aged 45–60 years with grade IV primary KOA diagnosed by an orthopaedic surgeon based on the Kellgren-Lawrence score participated in the study. The participants were equally and randomly divided into two groups (i.e. experimental and control), with 15 participants in each group. The participants in the control group performed conventional physiotherapy (CPT) that included ultrasound therapy, isometric quadriceps, SLR and isometric hip adductor strengthening exercises. The participants in the experimental group received ESWT in addition to CPT. Lower extremity functional scale (LEFS) score was measured before and after the four weeks of intervention. In both groups, a statistically significant (p = 0.001) improvement in LEFS was observed. In the experimental groups, it improved by 81.92% and in the control groups by 48.15%. A statistically significant (p < 0.001) difference was observed in LEFS post-intervention values between both groups. As demonstrated by our trial results, the addition of ESWT to the CPT program will yield beneficial results in ameliorating the functional disability in patients with primary KOA (grade IV). Further studies are needed to confirm and apply these findings to a larger cohort.

## Introduction

Knee osteoarthritis (KOA) is a progressive degenerative disease that is the most prevalent kind of arthritis that prevails worldwide, imparting a socio-economic burden on society^1,2^. Even though it has been strongly associated with ageing, with the most affected individuals over 50 years of age, that is not the primary cause behind this functional joint disorder^3^. As a polymorphic disorder, the exact pathogenesis of KOA is poorly understood; however, clinical data regarding its development supports the hypothesis that the synovial membrane, as well as the sub-chondral bone, are the key structures involved in the disease. Additionally, any deterioration in the joint components (joint capsule, ligaments, synovial membrane, and the surrounding muscles) can negatively impact the functioning of the joint, subsequently leading to its damage. The clinical manifestations of KOA include joint tenderness, pain, inflammation (varying degrees), crepitus and limited motion^4^.

The KOA has been categorized under primary (idiopathic in origin) and secondary, with the latter being associated with post-injury, inflammatory, infectious, dysplastic, and biochemical in origin^5,6^. The gold standard to confirm its diagnosis remains the radio-graphic demonstration, and in 1957, Kellgren and Lawrence (KL) were the first to lay the foundation for the standardized radio-graphic categorization scheme^7^. After that, KL classification for OA based on X-ray findings is the most extensively applied clinical diagnostic tool^8^. The KL division is based on the radiographic findings and grading system, which involves the allocation of the grade from 0 to 4 corresponding to the progression of the disease, with grade 0 implying the absence of OA while grade 4 implying a severe form of the disease. In addition, the KL division-based grades of severity (from 0 to 4) are related to the presumed sequential appearance of osteophytes and joint space loss^9,10^.

The current line of treatment for KOA incorporates surgical, pharmacological, and non-pharmacological interventions^11^. However, how efficient is the medicinal use to withstand the impact of OA is debatable, and the long-term consumption of these drugs has been associated with their adverse effects^12^. Consequently, there has been no ideal treatment for KOA so far. Therefore, the first line of treatment is typically non-invasive in the early phases of OA, given there is an absence of apparent lesions, deformities or abnormalities that require surgical intervention^13^. Therefore, physicians and physical therapists aim to alleviate pain, improve functional limitations, and improve quality of life while minimizing adverse reactions through a combination of therapeutic approaches^13^.

In the past few years, the need for knee replacement surgeries has substantially increased; however, it has been indicated that through the delivery of physical therapy in the nascent stages of KOA, this could be potentially reduced^14^. Extracorporeal shockwave therapy (ESWT) is one such non-invasive physical therapy intervention which emerged in the recent past to address the upswing of OA^15^. The mechanism behind this modality lies in the action of its acoustic shortwaves, which are quick, short in duration and have the capability to carry energy and disseminate it through the tissues and target area of treatment to produce physiological changes within them^16,17^. Zhao et al. were the first researchers in the world to assess and favor the impact of shockwave therapy for treating KOA and reported no adverse effects associated with this mode of intervention. This has encouraged others to conduct similar investigations utilizing ESWT as a potential treatment^18^. Since then, a growing number of clinical investigations have attempted to evaluate the efficacy of ESWT on the development of OA disease and cartilage degeneration^19–22^.

The exact physiological mechanism responsible for the benefits of ESWT is unknown, however, its clinical applications have been expounded and accredited to the following factors: the ESWT slows down the structural changes in the subchondral bone, thereby reducing the progressive deterioration of the cartilage^23^. Additionally, it has been suggested that ESWT delivers other potential advantages such as increased cell proliferation, protein biosynthesis, neovascularization, and pain inhibition. Integrating all the above processes has been thought to provide tissue restoration, pain amelioration and possible functional enhancement in the affected tissues^24^. On the grounds of existing evidence on ESWT, 14 studies (782 participants) were included in a recent systematic review with meta-analysis to evaluate the impact of this modality on the pain and functional status of individuals with KOA. This systematic review, which was conducted by Avendano-Coy et al. concluded that ESWT is an efficient and superior mode of treatment for individuals with (mild and moderate) KOA for the improvement in pain and functional status in the short-term ($\le$ 12 weeks) as compared to other conservative approaches. However, the review also reported moderate evidence of 'certainty' and a few minor side-effects regarding its use^12^. All 14 studies included in the Avendano-Coy et al.^12^ systematic review involved participants with less than grade IV KOA. However, there were only two studies that enrolled patients with grades III and IV and grade II–IV KOA. In these two studies, Shenouda et al. reported a positive impact of ESWT on reducing knee pain and improving functional status, while the latter by Imamura et al. did not find any improvement in the disabling knee pain due to primary OA after the application of shockwave therapy^25,26^.

Given the paucity of evidence and the contradicting results between the available evidence^25,26^, more research is required to further investigate the efficacy of ESWT on grade IV KOA. Thus, the present study aimed to examine the effects of ESWT on functional ability in patients suffering from grade IV KOA. We hypothesized that there would be a significant improvement in functional ability when using ESWT in patients with grade IV KOA. Considering that the current widely used treatment method for KOA grade IV is joint replacement surgery, investigating the hypothesis of this study might support the use of ESWT with those patients and reduce the complications related to surgical replacements and the subsequent economic burden.

## Materials and methods

### Participants

Thirty volunteers aged 45–60 years with grade IV primary KOA diagnosed by an orthopaedic surgeon based on the Kellgren-Lawrence score participated in the study. They were recruited from various government and private hospitals around Calicut city. The sample size was calculated as 30 by G*Power software (3.1.94) based on a previous study^27^, which showed a significant difference in the lower extremity functional scale (LEFS) score following hip strengthening exercise. The following parameters were used for the sample size calculation: mean differences between matched pairs, types of power analysis—A priori: compute require sample size – given α = 0.05, Power (1–ß err prob) = 0.95, and effect size = 1.23. The primary investigator screened the participants based on specific inclusion/exclusion criteria.

Participants with secondary KOA, rheumatoid arthritis, psoriatic arthritis, the presence of any red flags (metastatic growth, thrombophlebitis), neurologic or psychiatric impairments, and soft tissue injuries of the knee joint were excluded from the study. Patients who had undergone previous knee joint replacement or other surgeries in the knee joint, already received ESWT, Intra articular injection and patients who lost independent walking ability were also excluded.

### Procedure

**Figure 1:**
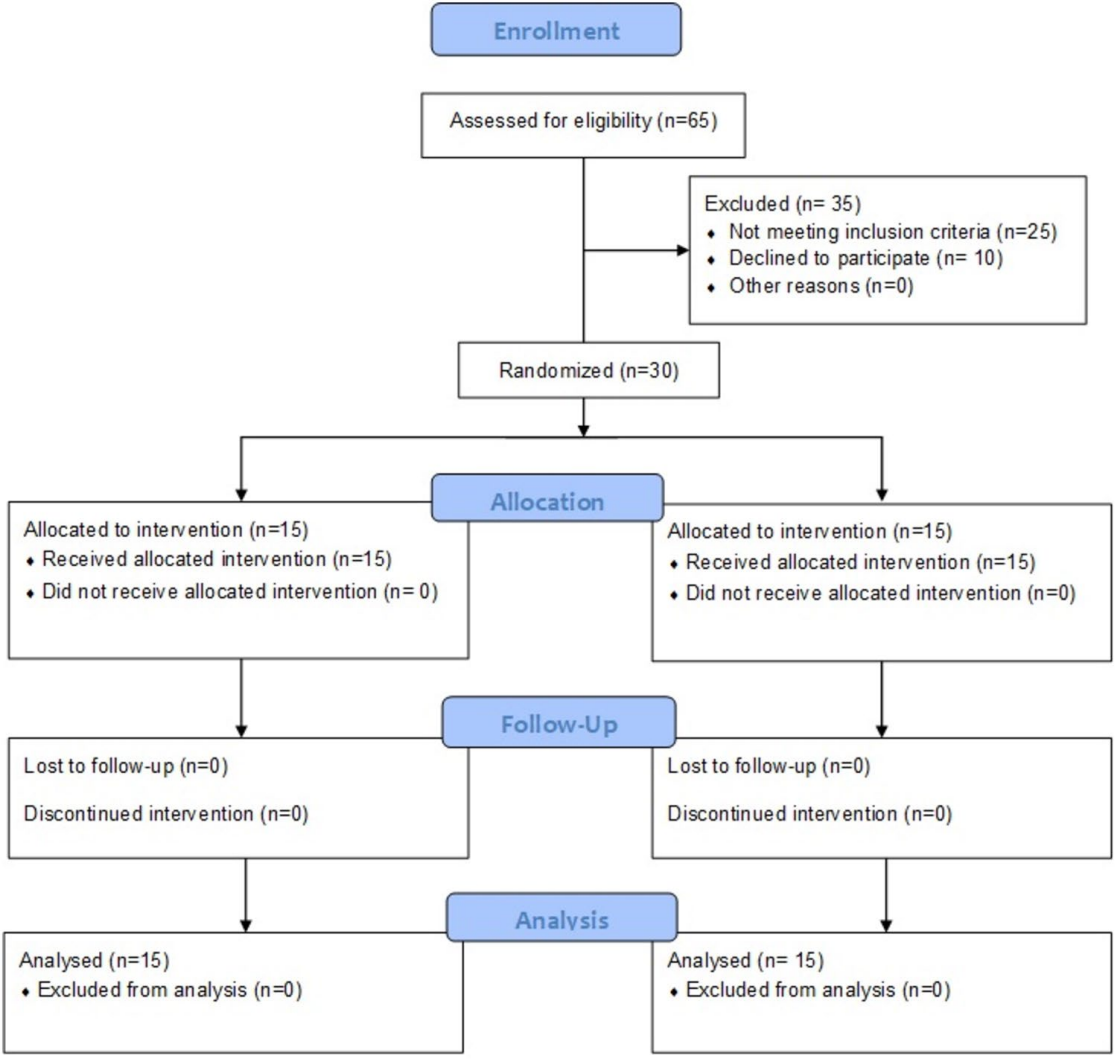
Consolidated standards of reporting trials (CONSORT) flow chart showing the numbers of participants assessed, recruited, randomization and analyzed during the study.

This randomized controlled trial adopted a two-group pretest–posttest experimental design. A familiarization session was conducted before the data collection to ensure the participants were acquainted with the procedure. An independent researcher not associated with this study performed randomization using statistical software SPSS (version 23) and the lottery method and randomly assigned the participants to the experimental (n = 15; 8 males, 7 females) and control groups (n = 15; 5 males, 10 females). Figure 1 shows the number of included and excluded participants. The outcome assessor was kept blinded to the study. The experimental group received ESWT in addition to conventional physical therapy (CPT), whereas the control group received the CPT alone. The baseline measurement was taken on the first session of the study before the first intervention session, and the post-test data was taken after four weeks of intervention. The participants were instructed to continue their regular activities and avoid extra exercises. The institutional ethical committee of AWH Special College approved the study (approval number: AWH/EC/03/2021/2), and all the procedures were done according to the declaration of Helsinki. The procedures were conducted at the physical therapy laboratory of AWH Special College. All procedures were performed in accordance with the relevant guidelines. Details of the protocol were given to the participants before starting any intervention. The participants agreed voluntarily and gave written informed consent. The study had been registered retrospectively in the protocol registration and results system (ClinicalTrials.gov) with ID: NCT06181955 on 26/12/2023.

### Intervention

#### Conventional physical therapy

CPT consisted of ultrasound therapy (UST) and three types of knee-strengthening exercises. The UST was performed once a day with an intensity of 1.5 watts/cm2 for seven minutes in continuous mode at the tender point around the knee joint based on the examination of the primary investigator. The strengthening exercises consisted of quadriceps sets, straight leg raising and isometric hip adduction exercises.

1. Quadriceps sets: While the participants were supine lying, a towel roll was placed beneath the knee. The participants were instructed to activate their thigh muscles maximally, press the towel roll with a straightened knee and hold the contraction for 5 seconds.
2. Straight leg raising (SLR) exercise: While the participants were in a supine lying position, they were instructed to perform a maximal isometric quadriceps contraction and lift the leg to 10 cm above the plinth with knee extension and hold the contraction during the lifting phase for 10 s.

##### Isometric hip adduction exercise

While the participants were supine, a small pillow was placed between the knees. The participants were instructed to press the pillow between the knees as strongly as possible and hold the contraction for five seconds. All exercises were performed in sets of 10 repetitions, with one set of all exercises performed twice daily. The primary investigator supervised all the treatment sessions.

#### Extracorporeal shockwave therapy (ESWT)

The ESWT was performed by an ESWT instrument (Chattanooga) once a week for four consecutive weeks (a total of four sessions). A radial mode of shockwave was selected for the treatment. The participant's skin was washed, and the hair was removed from the treatment site. While the participants were supine lying, with the target knee flexed at 90º, the physical therapist was positioned in the ipsilateral to the treatment side. The physical therapist determined the tender points of the target knee by palpation and the patellofemoral and tibiofemoral borders. An aqua sonic gel was applied before the delivery of ESWT to minimize the loss of shock waves at the interface between the applicator and skin. Two thousand pulses of 8-Hz frequency at 2.5 bars of pneumatic pressure were given to the participants in each session^25,28^.

### Outcome measurement

LEFS assessed the functional disability of the participants. It is a patient-reported outcome measure to assess the functional status of individuals with musculoskeletal conditions affecting the lower extremities. The scale consists of 20 items with a score ranging from 0 to 4 for each item, with scores of 0 representing extreme difficulty and 4 representing no difficulty, representing a higher level of functioning. The rationale behind using LEFS stemmed from its high reliability, validity and responsiveness to determine lower extremity functioning in patients with hip or KOA^29^.

### Data analysis

All statistical analysis was done using IBM SPSS software version 23. Baseline values of dependent variable LEFS were first examined for normality using the Shapiro–Wilk test of normality. This test revealed no normal distribution (p < 0.05) of baseline values of the LEFS variable in the control group. Therefore, non-parametric tests were used for further with-in and between-group analysis. The Wilcoxon Signed Ranks Test was used for with-in-group analysis, and Mann–Whitney *U* was performed for between-group analysis. The confidence interval was 95%, and p- p-values ≤ 0.05 were considered significant.

## Results

Data from thirty participants (15 in each group) were analyzed. The demographic data and baseline measurements of the participants from the experimental and control groups are available in Table 1. There was no significant difference in age, body mass, height, or BMI (p > 0.05). A statistically significant (p = 0.001) improvement was observed in the dependent variable (LEFS) in the experimental group. The mean value improved from 19.53 to 35.53. The dependent variable improved by 81.92%. A statistically significant (p = 0.001) improvement was also observed in LEFS in the control group. The mean value improved from 13 to 19.26. The dependent variable improved by 48.15%. A statistically significant (p < 0.001) difference was observed in LEFS post-intervention values. Greater improvement was observed in the experimental group.

**Table 1:** Participants’ characteristics and baseline measurement of both groups.

Characteristics	Experimental group	Control group
Age (years)	52.53 ± 5.35	53.53 ± 5.29
Height (cm.)	162.80 ± 4.17	165.48 ± 5.05
Weight (Kg)	61.67 ± 7.63	71.04 ± 13.16
BMI (kg/m^2^)	23.18 ± 2.60	25.77 ± 4.01
LEFS score (n)	13 + 8.09	19.53 + 5.19

Values are mean ± SD. *LEFS* lower extremity functional scale, *BMI* body mass index.

## Discussion

The pathophysiology and the treatment of KOA remain highly obscure^30,31^. The common complaints of the affected individuals are often debilitating pain and physical restrictions during the course of the disease^32,33^. Even though several clinical interventions have been applied to those seeking relief, none of those therapeutic approaches, both pharmacological and non-pharmacological, have successfully and entirely addressed the concerns associated with KOA^34–37^. Furthermore, the management of the chronic comorbidities (diabetes, anxiety, obesity, hypertension, etc.) that have been associated with OA requires a great expenditure. It places a socio-economic burden on the affected individuals and their communities^38^. As a result, there has been no standardized treatment or cure for this common orthopaedic condition, which affects millions of people, and the therapy goal of health professionals is to minimize the impact of pain and physical limitations while simultaneously aiming towards improving the quality of life.

Therefore, the present study aimed to examine the impact of the ESWT using a clinical trial for individuals with grade IV KOA, on the functional status parameter. We hypothesized that there may be a significant improvement in functional ability with ESWT in patients of grade IV KOA. The results following the four weeks of study protocol demonstrated that ESWT with CPT and CPT alone were effective in improving functional ability in patients of grade IV KOA. There was a statistically significant difference (from pre- to post-mean value) on the outcome measure (LEFS) in the experimental group with an improvement of 81.92%, while the significant improvement recorded for LEFS in the control group was 48.15%. Therefore, the experimental group that received ESWT with CPT showed greater improvement than the control group (Table 2).

**Table 2:** Pre- and post-intervention data of both groups.

Group	Pre	Post	Mean difference	t-value	p-value
Experimental	13 ± 8.09	32.73 ± 3.31	6.74	13.67	p < 0.05*
Control	19.53 ± 5.19	36.07 ± 3.51	4.33	20.56	p < 0.05*

*Significant.

The results of the present study are in agreement with other comparable RCTs. A study by Lizis et al. investigated the effectiveness of ESWT on patients with KOA and reported an improvement in the physical function in those patients^28^. Similarly, El- Sakka et al. reported the beneficial impact of shock wave therapy in enhancing the functional performance in patients with primary OA^39^. However, both of these studies included patients who were diagnosed with a lower grade (grade I-III) of OA on the Kellgren-Lawrence scale. Therefore the present study established the potency of ESWT in those with a severe form of KOA (grade-IV). In addition, there were no dropouts in the present study, which might have impacted the validity of results. In the present study, the shockwave therapy probe were held on the most painful areas (as determined by the trained physical therapist through palpation) and on the patellofemoral and tibiofemoral borders of the affected knee as the effects of ESWT might be site-specific^18,40^. Unlike other studies that used WOMAC (Western Ontario and McMaster universities osteoarthritis index), the present study used LEFS to assess functional status as it is a more appropriate measure to evaluate lower extremity function in patients with KOA along with a better discriminant validity compared to the WOMAC^41^. The present study also provided participants in both groups with a CPT (UST and three types of knee strengthening exercises), which might have reduced knee pain and improved functional ability in the participants^42,43^. Thus, the present study may guide future clinical trials towards informed decision-making considering the above parameters to observe the long-term effects of ESWT in a larger population with KOA.

The major characteristics of KOA have been attributed to the deterioration of articular cartilage and the remodeling of subchondral bone^44^. It is hypothesized that a dysbiotic gut microbiome causes microbial products or metabolites to leak through an inadequate epithelial barrier, hence causing or aggravating pain associated with osteoarthritis. This can lead to a localized and systemic inflammatory state^45^. ESWT is a non-invasive modality that utilizes mechanical stimulation and has been implemented for treating KOA in recent years^15^. Even though the exact mechanism behind its therapeutic impact remains ambiguous, it has been applied in several clinical trials and animal model studies, which have demonstrated the potential benefits of this intervention on cartilage, subchondral bone, and the adjacent muscles and tissues^15,44,46^. Moreover, to examine its impact, several studies have compared ESWT with alternative modes of treatment, namely, eccentric training, hyaluronic acid (intra-articular) injections, and kinesiotherapy, and indicated that shock wave therapy can be safely applied in the treatment of KOA^28,47,48^. Notarnicola et al. confirmed the potential of ESWT through an in vitro cell experiment. They reported that ESWT increased the expression of IL-10 (responsible for inhibiting the production of pro-inflammatory cytokines). In contrast, it decreased the expression of both N-cadherin and b-catenin (their upregulation is responsible for the degeneration)^49^. Further, knee pain as a sequela of OA is a primary complaint of the affected individuals; in several cases, the pain becomes difficult to subside with even medications and non-pharmacological interventions^50–52^. The application of ESWT for pain management has also demonstrated a significant reduction in pain through the downregulation of the release of P substance^53^, expression of peptide associated with pain-related calcitonin gene in dorsal root ganglion^54^ and through the suppression of pain pathway (production as well as propagation), by acting on peripheral nerve endings.

Finally, in our study, the greater improvement in the experimental group that received ESWT over the control group can likely be attributed to shockwave’s effects, which cause microbreaks in avascular or impaired vascularized tissues, thereby inducing revascularization and stem cell growth. This primary physiological advantage of shockwaves over the US was explained in another similar study^28,55^. Overall, we found no adverse reaction to ESWT in our study, which is also consistent with previous trials. It is clear that in the early stages of KOA (KL grade I, II and III), healthcare professionals’ resort to non-surgical interventions and have found it quite useful to address the complications of the disease; however, knee replacement is the most effective method for those who are in the advanced stage of the disease (KL grade IV). Therefore, the presented study aimed to examine the effectiveness of ESWT for improving the functional ability of grade IV KOA. The findings of our trial demonstrated that the shock wave and the CPT have a beneficial effect in treating primary KOA as a non-invasive modality for improving clinical and functional performance.

### Limitations

There are several limitations to our study. We included patients with KOA of primary origin; therefore, it is ambiguous if similar results could be achieved in patients with secondary KOA. An Ultrasonography or MRI investigation was not carried out before and after the treatment, which could have displayed more detailed results regarding the effectiveness of ESWT. Similarly, an x-ray investigation was performed after the completion of the study protocol, which could provide a more detailed conclusion regarding the impact of shortwaves on the target joint. Our study did not incorporate objective assessments other than LEFS, as functional limitation is the major concern in the advanced stage of OA. Previous studies have reported that even though pain is a symptom, functional limitations overshadow pain in the advanced stages of OA. The study was conducted on a small sample size for a short duration, and a follow-up period was not performed. Therefore, further trials are required to assess the sustained impact of ESWT with longer study duration in a more diverse study population. In addition, the experimental group reported lower scores in weight, BMI, and LEFS. However, these differences are not statistically significant. It is unknown whether these differences potentially influenced the study outcome.

### Clinical implication and future direction

The study shows significant improvements in the functional ability of patients with grade IV KOA, suggesting that ESWT can be a valuable treatment option for patients. ESWT may help to enhance mobility and daily functioning, thereby improving a patient's quality of life. As a noninvasive treatment, it could be more cost-effective compared to surgery and, due to its effects, can reduce the need for surgical interventions. ESWT may also be integrated along with other treatments currently available for severe KOA to receive a better outcome.

Since the intervention of ESWT was given only for 4 weeks in the present study, conducting long-term studies along with multiple post-treatment follow-ups would be beneficial in determining the durability of ESWT. ESWT can also be compared with advanced treatment options like platelet-rich plasma (PRP) injections or hyaluronic acid injections to assess relative benefits. Combining ESWT with these treatments may also provide favorable results. Expanding the study by including larger and more diverse populations of patients can be considered in upcoming studies so that the generalizability of results can be ensured.

The outcome score used in the study, LEFS, is a self-reported questionnaire that only gives a subjective assessment of treatment outcome. Hence, future studies can also aim to include imaging techniques like X-rays, ultrasonography, or MRI, which will provide a better overview of any changes that have occurred in the overall joint structure post-treatment with ESWT. Future studies may also explore the inclusion of gait analysis and functional performance tests to assess if ESWT can give more impactful results.

## Conclusion

As demonstrated by our trial results, the addition of extracorporeal shockwave therapy (ESWT) to the conventional physical therapy (CPT) will yield beneficial results in ameliorating the functional disability in patients with primary KOA (grade IV). Further studies are needed to confirm and apply these findings in a larger cohort.

## Data Availability

The data associated with the paper are not publicly available but are available from the corresponding author on reasonable request.
